# Lipidomics analysis of human follicular fluid form normal-weight patients with polycystic ovary syndrome: a pilot study

**DOI:** 10.1186/s13048-021-00885-y

**Published:** 2021-10-13

**Authors:** Yanna Ban, Haiying Ran, Ying Chen, Li Ma

**Affiliations:** 1 Reproductive Medicine Center, The First Affiliated Hospital of Chongqing Medical University, Chongqing, 400016 P. R. China; 2 Biomedical Analysis Center, College of Basic Medical Sciences, Army Medical University, Chongqing, 400038 P. R. China; 3 The Chongqing Key Laboratory of Translational Medicine in Major Metabolic Diseases, The First Affiliated Hospital of Chongqing Medical University, Chongqing, 400016 P. R. China; 4 Laboratory of Lipid &Glucose Metabolism, The First Affiliated Hospital of Chongqing Medical University, Chongqing, 400016 P. R. China

**Keywords:** Polycystic ovary syndrome, Lipidomics, Follicular fluid, HPLC-MS

## Abstract

**Background:**

The polycystic ovary syndrome (PCOS) is the most common endocrine associated with insulin resistance, even in the absence of overweight. The global lipid profile of the follicular fluid in PCOS with normal weight as yet has not been investigated. The objection of this pilot study was to explore the changes of lipids in the follicular fluid of PCOS with normal weight.

**Methods:**

Follicular fluid samples were collected from patients who underwent IVF, including normal-weight women without PCOS (control group, *n* = 10) and normal-weight women with PCOS (PCOS group, *n* = 8). A lipidomic analysis was performed by high performance liquid chromatography/ mass spectrometry (HPLC-MS). Multidimensional statistical analysis was performed to disclose the global differences between the two groups. Further, differential lipid analysis between the two groups was performed by Fold Change Analysis (FC Analysis) and T-test to screen potential markers.

**Results:**

All 812 species of 32 subclasses of lipids were identified by lipidomics analysis. 108 kinds of lipids were considered as the potential candidate differential metabolites with the score of variable importance in the project (VIP) more than 1 by the orthogonal partial least squares discriminant analysis. 32 lipids were significantly different between the PCOS group and the control group simultaneously with FC > 1.5 or FC < 0.67, *p*-value < 0.05 and VIP value > 1. These differential species of lipid belong to lipid subclasses including triglycerides (TG), phosphatidylethanolamines (PE) and phosphatidylinositols (PI).

**Conclusion:**

The identified differential lipids in the follicular fluid may be considered as candidate biomarkers as well as therapeutic targets of PCOS with normal-weight.

**Supplementary Information:**

The online version contains supplementary material available at 10.1186/s13048-021-00885-y.

## Introduction

Polycystic ovary syndrome (PCOS) is one of the most common endocrine and metabolic disorders with a prevalence ranging from 5 to 10% among women of reproductive age. The clinical manifestations include menstrual irregularities, anovulatory infertility, signs of androgen excess, and metabolic and psychological disorders. POCS is the most important cause of anovulation infertility, with a high incidence of 18% [1]. In addition, the presence of low oocyte quality is common in PCOS patients undergoingin vitro fertilization (IVF) treatment [2]. Follicular fluid (FF) serves as acomplex microenvironment for oocyte growth, follicular maturation, and germ cell-somatic cell communication [1]. It accumulates all metabolisms during oocytes growth. Hence, the changes in the metabolites of FF have been linked to impaired oocyte quality and outcomes of IVF in patients with PCOS. Metabolomics is a high-throughput approach for the detection of extensive small-molecule metabolites in various biological samples that can provide useful information related to diagnostic biomarker and pathogenesis mechanism of diseases. Currently, metabolomics analysis of FF has revealed that lipid metabolites are significantly changed in PCOS patients [3, 4]. Lipidomics is one of the metabolomics approach that focuses on lipids and is a promising technique for overviewing lipid profiles in body fluid, blood and tissues. Currently, there are a few published lipidomics studies on PCOS in plasma [5, 6]. However, the lipidomics of PCOS in FF, which directly reflects the oocyte micro-environment, has not been investigated previously. 

Women with PCOS are often overweight or obese. However, due to the diversity of clinical and biochemical manifestations of PCOS, 30–50% of patients are with normal weight [7]. The presence or absence of obesity is one of the most important factors influencing PCOS phenotypes. The incidence of insulin resistance and metabolic syndrome was found to be higher in obese PCOS patients than in non-obese PCOS patients, but the latter also had metabolic abnormalities [8]. A previous study found that non-overweight women with PCOS had higher luteinizing hormone (LH) and follicle-stimulating hormone (FSH) levels in plasma than healthy women. Moreover, basal insulin, total cholesterol, low-density lipoprotein (LDL), very low-density lipoprotein (VLDL), and triglyceride levels in the overweight group were found to be significantly higher. However,  there were no differences  in triglyceride and total cholesterol in plasma between the normal normal-weight women with PCOS and normal-weight women without PCOS [9]. To date, the lipids in FF from normal-weight women with PCOS have never been studied. In the present pilot study, we used lipidomics technology based on high performance liquid chromatography/ mass spectrometry (HPLC-MS) platform to obtain a comprehensive picture of the lipid alterations that occur in the FF of PCOS patients with normal weight.

## Experimental

### Subjects

We designed a case-control pilot study, including FFs from 8 women with normal weight (body mass index [BMI] < 25 kg/m^2^) who were diagnosed with PCOS and 10 healthy women. This study was performed at the First Affiliated Hospital of Chongqing Medical University and wasconducted in accordance with the ethics guidelines of the Declaration of Helsinki. The FFs were collected  from the patients  who underwent IVF or intracytoplasmic sperm injection (ICSI) in our reproductive centre between January 2019 and December 2019. Written informed consents were obtained from all subjects, and all the experimental protocols were approved by the ethics committee of the First Affiliated Hospital of Chongqing Medical University. The enrolment criteria for PCOS were based on the presence of any two or three features – hyperandrogenism, menstrual irregularity, and polycystic ovary morphology, according to the Rotterdam consensus criteria. None of the women had abnormal serum androgen hormone levels. The control group included women who sought treatment for tubal infertility or male factors with normal ovarian reserve (regular menstrual cycles, and anti-Müllerian hormone [AMH] concentration of ≥1.1 ng/mL) and normal BMI. Women with endometriosis, glucose metabolism, cancer, or other medical disorders that could affect folliculogenesis were excluded. The gonadotropin-releasing hormone antagonist protocol was used for controlled ovarian stimulation. When the follicle reached an average diameter of ≥18 mm, urinary human chorionic gonadotropin (u-HCG, Lizhu, Zhuhai, China) was administered, and ultrasound-guided FF samples were collected after 36 h using an 18-gauge single-lumen aspiration needle.

### Preparation of samples

Serum and plasma samples were collected between days 2 and 4 of the menstrual cycle in the control group and between 2 and 4 days after a spontaneous bleeding episode in patients with PCOS after an overnight fast. Circulating levels of hormones were measured by electrochemiluminescence assay (Cobas e602; Roche Diagnostics GmbH, Mannheim, Germany) and the biochemical indexes were measured by enzymatic method with an automatic biochemical analyzer (Cobas c701; Roche Diagnostics GmbH, Mannheim, Germany). Clear FF samples  without macroscopic blood contamination were included. After oocyte isolation, the pooled FF samples were centrifuged at 800×g for 10 min to remove particulates. The FF supernatant was stored at − 80 °C until analysis. Before analysis, the FF samples were thawed in a 4 °C water bath. Lipids were extracted using methyl tert-butyl ether (MTBE) method. Briefly, the samples were homogenized with 200 μL of water and 240 μL of methanol. Then 800 μL of MTBE was added and the mixture was ultrasound 20 min at 4 °C followed by sitting for 30 min at room temperature. The solution was centrifuged at 14,000×g for 15 min at 10 °C and the upper organic solvent layer was obtained and dried under nitrogen. The samples were reconstituted in 200 μL of 90% isopropanol/acetonitrile and centrifuged at 14,000×g for 15 min at 10 °C, and 3 μL of the sample was injected into  the LC-MS system. A pooled FF sample from all healthy controls and PCOS patients was used as quality control (QC) and underwent the same sample extraction procedures.

### Chemicals and reagents

MS-grade methanol, MS-grade acetonitrile and HPLC-grade isopropanol were purchased from ThermoFisher (Thermo Fisher Scientific Co., Waltham, Massachusetts, USA). HPLC-grade formic acid, HPLC-grade ammonium formate and MTBE were purchased from Sigma-Aldrich (Sigma Chemical Co., St. Louis, Missouri, USA).

### UPLC-MS experiments

The UPLC-MS experiments were performed using a Q-Exactive Plus mass spectrometer (Thermo Scientific) system equipped with a UHPLC Nexera LC-30A (SHIMADZU). Mass spectrometry performed in either the ESI positive or negative mode. ESI parameters were optimized and preset for all measurements as follows: Source temperature, 300 °C, Capillary Temp, 350 °C, the ion spray voltage was set at 3000 V, S-Lens RF Level was set at 50% and the scan range of the instruments was set at m/z 200–1800. Reversed phase chromatographic separation was performed on a Waters ACQUITY UPLC CSH C18 column (2.1 mm × 100 mm, 1.7 μm) maintained at 45 °C. Solvent A was acetonitrile–water (6:4, v/v) with 0.1% formic acid and 0.1 mM ammonium formate and solvent B was acetonitrile–isopropanol (1:9, v/v) with 0.1% formic acid and 0.1 mM ammonium formate. The initial mobile phase was 30% solvent B at a flow rate of 300 μL/min. It was held for 2 min, and then linearly increased to 100% solvent B in 23 min, followed by equilibration with 5% solvent B for 10 min. 

The samples were analyzed in a random order. To test the reproducibility of the sample preparation procedure and LC-MS analysis, QC samples were injected at the beginning of the run and after every eight real samples. Each of the prepared QC samples was analyzed only once.

### Data processing

The raw data were processed by LipidSearch software version 4.1 (Thermo Scientific™) for peak identification, lipid identification by MS/MS, peak extraction, peak alignment, and quantitative analysis. The parameters were set as follows: precursor tolerance: 5 ppm, product tolerance: 5 ppm, and product ion threshold: 5%. The extraction data was normalized by total peak area after removed the lipid with RSD > 30% among samples.

### Statistical analysis

The software simca-p 14.1 (Umetrics, Umea, Sweden) was used for pattern recognition. After pareto-scaling pretreatment, multidimensional statistical analysis was performed, including unsupervised principal component analysis (PCA) and orthogonal partial least square discriminant analysis (OPLS-DA). One-dimensional statistical analysis by SPSS Statistics v17.0.0 (SPSS, Inc., Chicago, USA) included Student’s t-test and variance multiple analysis. R software was used to draw the volcano map, hierarchical clustering analysis map and correlation analysis map. Comparisons of parameters between two groups were performed by independent-Sample T test or non-parametric test, depending on the distribution (normal or not) of the examined variables.

## Results

### Clinical characteristics

The selected clinical baseline characteristics ofthe participants in our study are summarized in Table 1. The patients and controls were well matched for age and BMI. There were no significant differences in LH, FSH, LH/FSH, E2 (Estradiol), T (testosterone) levels, blood glucose, cholesterol, triglyceride, high-density lipoprotein cholesterol (HDL-C), low-density lipoprotein cholesterol (LDL-C), fertilization rate, cleavage rate and embryo top-quality rate between normal-weight PCOS patients and controls. AMH and No. of oocyte retrieve in PCOS were significantly higher than the control group.

**Table 1:** Main clinical characteristics of the study groups

	Controls(*n* = ten)	PCOS(*n* = eight)	*P*-value
Age (years)	28.80 ± 2.74	27.12 ± 3.40	0.26
BMI (kg/m^2^)	20.96 ± 1.99	19.96 ± 1.77	0.28
LH (mIU/mL)	4.20 ± 2.61	5.57 ± 2.48	0.22
FSH (mIU/mL)	5.86 ± 1.64	6.66 ± 2.21	0.39
LH/FSH	0.76 ± 0.63	0.93 ± 0.49	0.28
E_2_(pg/mL)	44.3 ± 40.69	68.75 ± 39.91	0.22
T (ng/mL)	0.49 ± 0.14	0.53 ± 0.11	0.52
Glucose (mmol/l)	4.83 ± 0.42	5.19 ± 0.46	0.52
Cholesterol (mmol/L)	4.25 ± 0.25	4.12 ± 0.32	0.77
triglyceride (mmol/L)	0.87 ± 0.28	0.88 ± 0.40	0.96
HDL-C (mmol/L)	1.47 ± 0.28	1.56 ± 0.54	0.64
LDL-C (mmol/L)	2.40 ± 0.71	2.02 ± 0.41	0.17
AMH (ng/mL)	3.52 ± 1.12	12.97 ± 3.42	< 0.001*
No.of Oocyte retrieve	13.2 ± 5.59	22.3 ± 5.99	0.004*
Normal fertilization Rate(%)	87.03 ± 0.18	79.24 ± 0.16	0.40
Cleavage Rate(%)	95.08 ± 0.57	87.76 ± 0.11	0.07
Embryo top-quality Rate(%)	43.44 ± 0.21	53.42 ± 0.27	0.43

*is indicated *p* < 0.05 values. Data presented as mean ± standard deviation (SD)

Abbreviations: *BMI* body mass index; *FSH* follicle-stimulating hormone; *LH* luteinizing hormone; *E2* estradiol; *T* testosterone; *HDL-C* high-density lipoprotein cholesterol; *LDL-C* low-density lipoprotein cholesterol; *AMH* anti-Mullerian hormone

normal fertilization rate = no. oocytes with 2PN and 2 PB/no. COC inseminated× 100%; cleavage rate = no. cleaved embryos on Day 2/no. 2PN/2 PB oocytes on Day 1 × 100%; embryo top-quality rate = top quality embryos/no. normally fertilized oocytes× 100%.

### Quality control

**Fig. 1:**
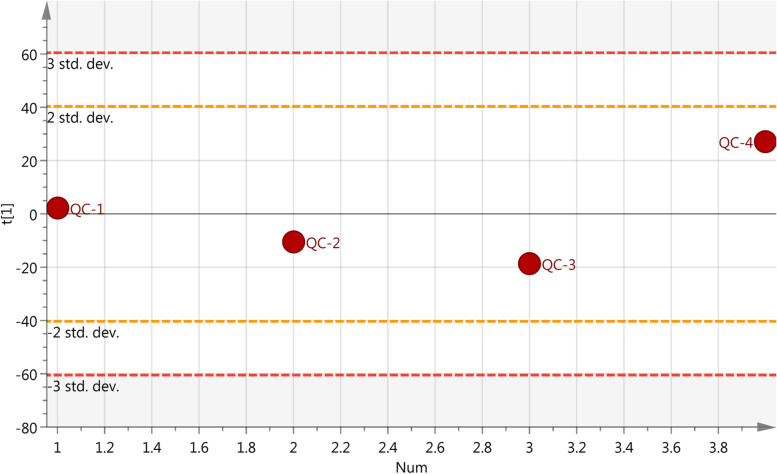
Quality control charts

Four QC samples were analyzed by both ESI+ and ESI- scan models, described in Section 2.4. Next, the base peak chromatograms were extracted and overlapped to compare the response intensity and the retention time of the peak. The results showed that the response intensity and retention time of the peak of each QC sample basically overlapped (shown in Figure 1 of the supplementary material). Pearson correlation analysis was then applied to the four QC samples of the response intensity of the extracted peaks. The results showed that the correlation coefficients were higher than 0.9 as shown in Figure 2 of the supplementary material. The peaks of all experimental samples and QC samples were extracted to perform PCA after Pareto-scaling. The results showed that the QC samples were closely packed together. A multivariate control chart was used to monitor the stability of the method. The Y-axis of the chart is the variance of the first principal component of the PCA model for all QC samples, and the X-axis is the loading order of the QC samples. The variance was no more than two standard deviations, indicating that the analysis system was stable and all the data were under control (Fig. 1). The relative standard deviation (RSD) of peaks in the QC samples is an important indicator of data quality. The ratio of peaks with an RSD of no more than 30% was more than 80% in the four QC samples. All these results indicated that the method had good repeatability, reliability, and stability for lipidomics analysis.

### Identification of lipid compounds

**Fig. 2:**
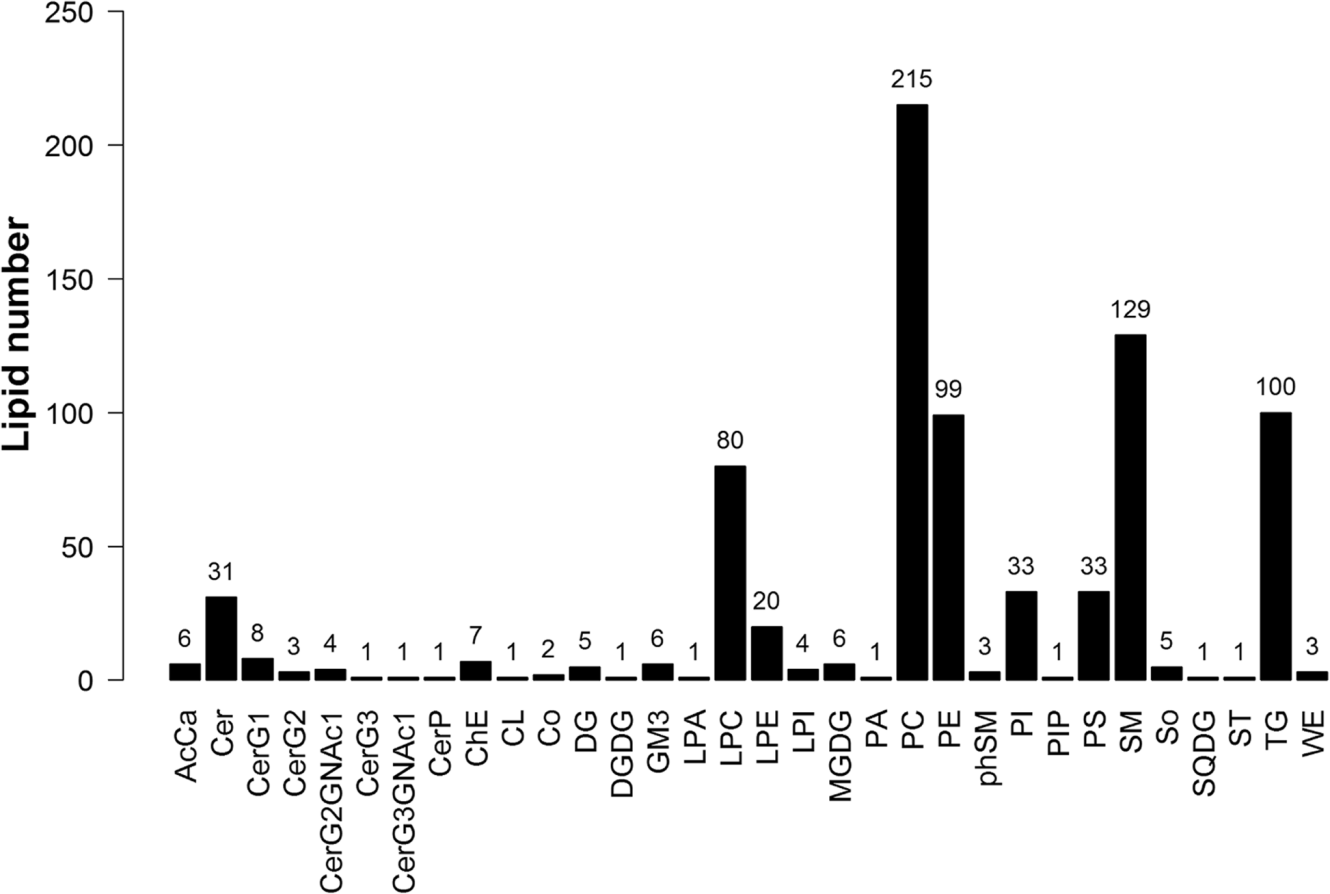
Classification of the identified lipids

Data obtained from the positive and negative scan models of HPLC–MS were analyzed by LipidSearch. All 812 species of 32 lipid subclasses were identified (Fig. 2). Thedifferences between the two groups in lipid subclasses are shown in figure 3 of the supplementary material.

### Data modeling

**Fig. 3:**
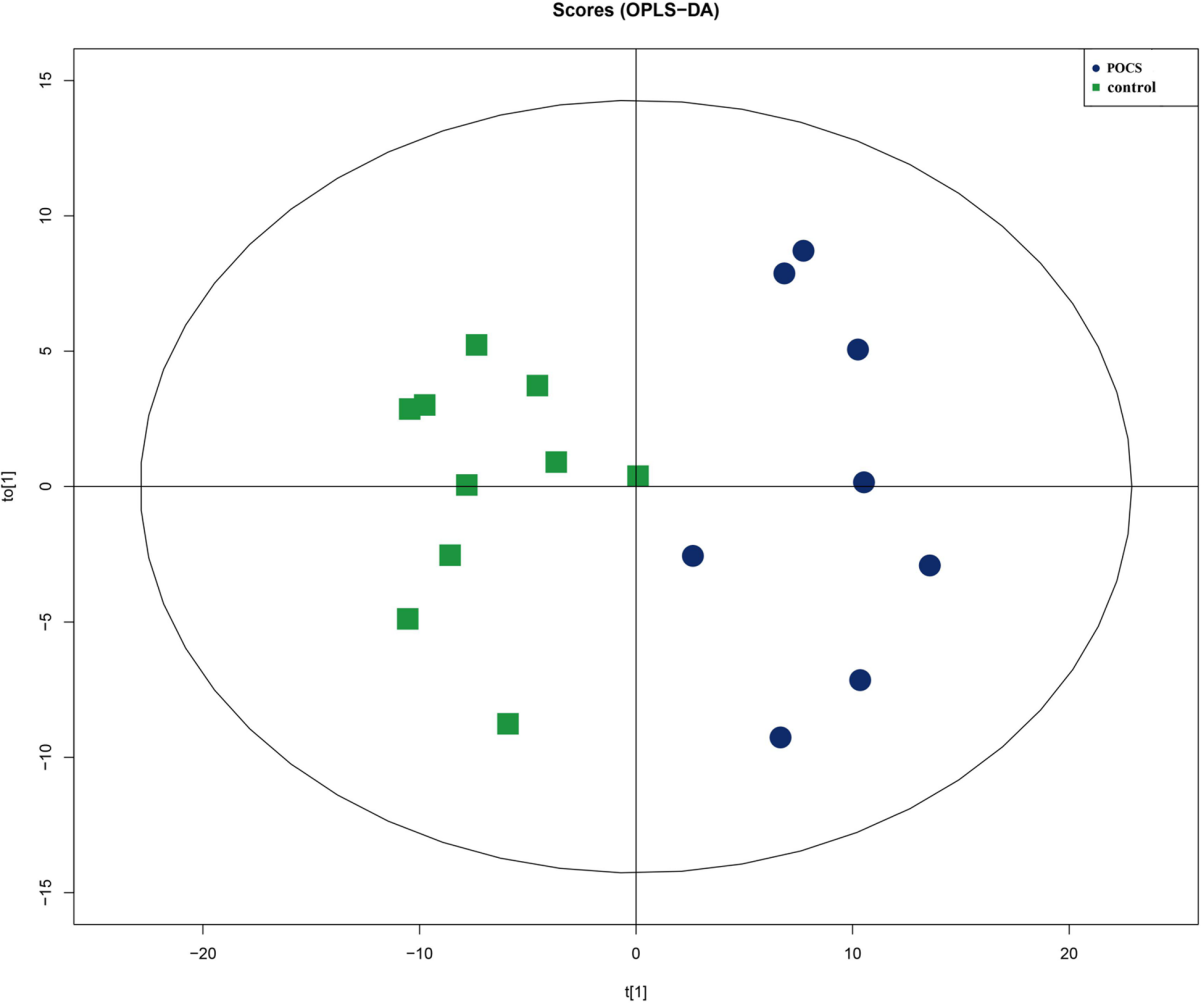
Score plots of OPLS-DA model showed a clear separation between normal weight PCOS women (blue circle) to control women (green square) with p (CV-ANOVA) = 2.3E-005

In this study, the PCA of FF was constructed to reveal the global differences of lipid between PCOS patients and healthy controls. In the PCA model, the clusters were not distinctly separated in the score plots, which might be due to the complexity and variation of clinical samples (shown in figure 3 of the supplementary material). The green box represents healthy controls, and the blue dot represents PCOS patients. OPLS-DA is a supervised method developed from PCA to remove unrelated noise from the predictive variables and obtain more reliable metabolite information. The two-dimensional scores of the OPLS-DA plot showed a clear separation between the healthy controls and PCOS patients with p (CV-ANOVA) = 2.3E-005. (Fig. 3).

### Identification of potential biomarkers

**Fig. 4:**
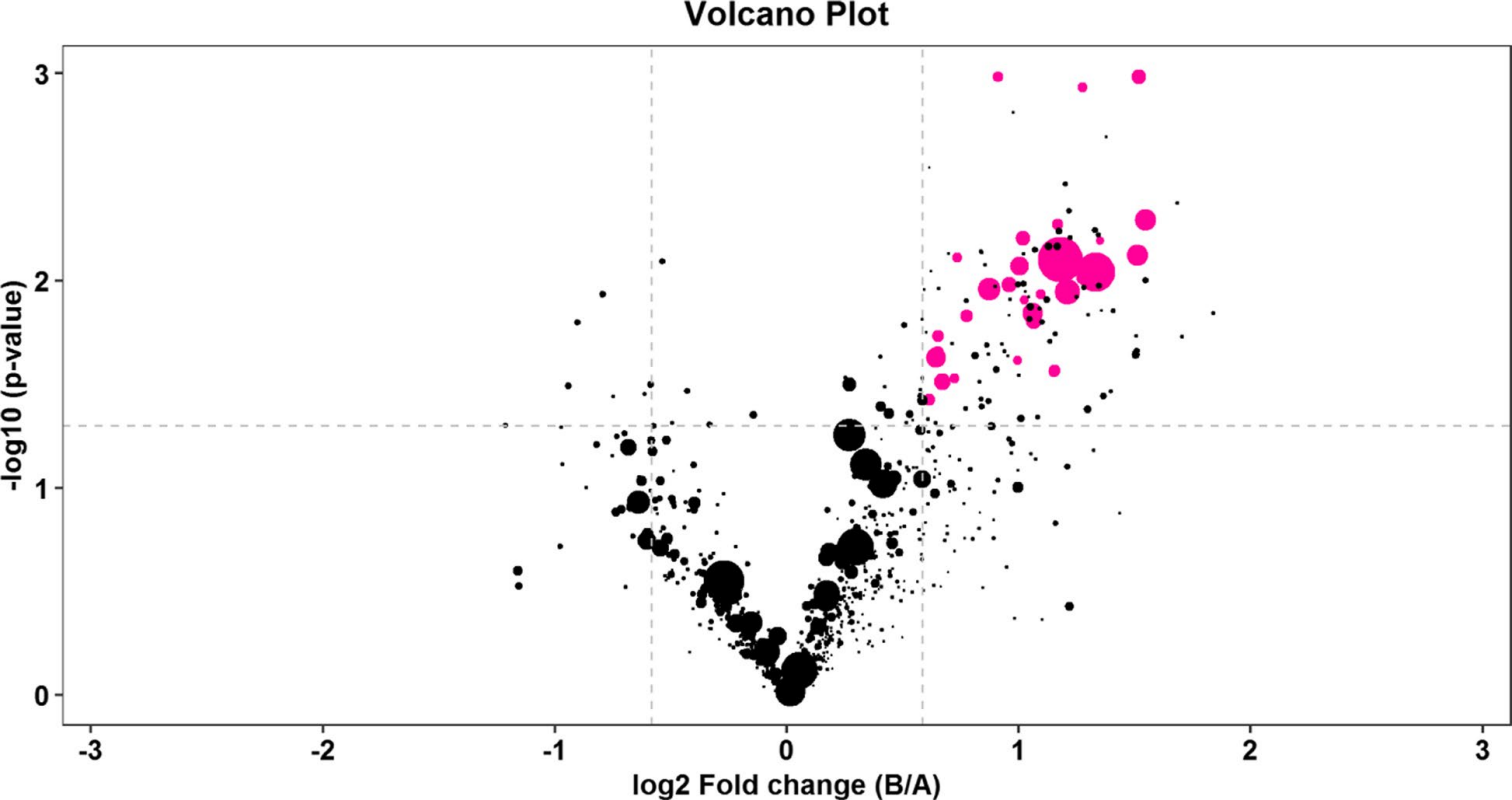
The volcano plot based on FC, VIP and *P* values of the differential lipid molecules. The purple dots represent the differential lipid molecules with FC > 1.5, *P* value < 0.05 and VIP value > 1 simultaneously. The area of the dots represents the VIP valve

Potential differential metabolites were defined according to the variable importance in projection (VIP) values based on OPLS-DA. Usually, lipid molecules with VIP ≥1 are considered to have contribute significantly to c model interpretation. As a result, 108 typesof lipids were considered as  potential candidate differential metabolites with VIP scores more than 1. Among these lipids, 36 were significantly different between the PCOS group and the control group analyzed by T-test/non-parametric test (*P* < 0.05), as shown in Table 1 of the supplementary material. The differential lipids between the two groups were performed by Fold Change Analysis (FC Analysis) and T-test to screen potential biomarkers. The screening criteria is FC > 1.5 or FC < 0.67 and *P* value < 0.05. The information of the differential expression multiples, *P* values and VIP values of lipid molecules were displayed in  a volcano plot, as shown in Fig. 4. The purple dots in the volcano plot are the differential lipid molecules that are simultaneously with FC > 1.5, *P* value < 0.05 and VIP value > 1.The area of the dots represents the VIP valve. The larger the bubble area  shown in the plot, the greater the VI*P* value. As a result, there are 32 lipids with FC > 1.5, P value < 0.05 and VIP value > 1 simultaneously Table 2).

**Table 2:** The candidate lipids which as potential biomarkers to separate normal weight PCOS women to control women

LipidIon	Class	Ion Formula	CalMz	RT-(min)	Fold Change	***P***-value	VIP
PE(16:0/22:6) + H	PE	C43 H75 O8 N1 P1	764.522484	10.2352508	2.1364297	0.01170255	1.2827738
TG(16:0/14:0/18:1) + NH4	TG	C51 H100 O6 N1	822.754516	20.7435549	2.82786013	0.00768395	1.04024184
TG(16:1/16:1/18:1) + NH4	TG	C53 H100 O6 N1	846.754516	19.714067	2.85935032	0.00103754	2.07802736
TG(16:0/16:0/18:2) + NH4	TG	C53 H102 O6 N1	848.770166	20.8812114	2.84645308	0.00753119	3.11342089
TG(16:0/16:0/18:1) + NH4	TG	C53 H104 O6 N1	850.785816	21.7834094	2.9164798	0.00508461	3.14637718
TG(16:1/18:1/18:2) + NH4	TG	C55 H102 O6 N1	872.770166	19.859873	2.51669633	0.00941933	4.83457578
TG(16:0/18:1/18:2) + NH4	TG	C55 H104 O6 N1	874.785816	20.8954062	2.26683859	0.00796182	7.04217237
TG(16:0/18:1/18:2) + NH4	TG	C55 H104 O6 N1	874.785816	19.8521076	2.42105627	0.00117044	1.21618584
TG(16:0/18:1/18:1) + NH4	TG	C55 H106 O6 N1	876.801466	21.7866945	2.51369634	0.00910841	6.11421082
TG(18:0/16:0/18:1) + NH4	TG	C55 H108 O6 N1	878.817116	22.584629	2.22068302	0.0275356	1.3333522
TG(18:1/18:2/18:3) + NH4	TG	C57 H102 O6 N1	896.770166	19.4825832	2.55170831	0.00642044	1.03093598
TG(18:2/18:2/18:2) + NH4	TG	C57 H102 O6 N1	896.770166	18.8771548	2.24487784	0.00535694	1.51541463
TG(16:0/18:1/20:4) + NH4	TG	C57 H104 O6 N1	898.785816	20.5420673	2.42182935	0.00972723	1.68243907
TG(18:1/18:2/18:2) + NH4	TG	C57 H104 O6 N1	898.785816	19.884727	2.00402129	0.00849049	2.6606902
TG(18:1/18:1/18:2) + NH4	TG	C57 H106 O6 N1	900.801466	20.9160682	1.83056246	0.01101498	3.33870883
TG(18:0/18:1/18:2) + NH4	TG	C57 H108 O6 N1	902.817116	20.8947311	1.52937154	0.03751919	1.62092888
TG(18:0/18:1/18:2) + NH4	TG	C57 H108 O6 N1	902.817116	21.8017114	2.30938518	0.01129426	3.83179623
TG(16:0/18:1/20:1) + NH4	TG	C57 H110 O6 N1	904.832766	22.5836836	2.22447076	0.02719304	1.57736708
Cer(d18:1/16:0) + HCOO	Cer	C35 H68 O5 N1	582.510298	11.3804774	1.65187002	0.02982499	1.19827027
PE(16:0/18:2)-H	PE	C39 H73 O8 N1 P1	714.507931	10.7122963	2.02509893	0.00623164	2.06847352
PE(16:0/18:1)-H	PE	C39 H75 O8 N1 P1	716.523581	11.4653225	1.87622225	0.00103417	1.36389091
PE(16:0/20:4)-H	PE	C41 H73 O8 N1 P1	738.507931	10.5345784	1.94530888	0.01049121	2.08653155
PE(18:0/18:2)-H	PE	C41 H77 O8 N1 P1	742.539231	11.6753321	1.59339478	0.03091878	2.34017355
PE(18:0/18:1)-H	PE	C41 H79 O8 N1 P1	744.554881	12.413823	1.66288434	0.00777766	1.2000378
PE(16:0/22:6)-H	PE	C43 H73 O8 N1 P1	762.507931	10.2584914	2.08517498	0.01442886	3.08345178
PE(18:1/20:4)-H	PE	C43 H75 O8 N1 P1	764.523581	10.6214751	1.71023624	0.0148648	1.68604552
PE(16:0/22:5)-H	PE	C43 H75 O8 N1 P1	764.523581	10.9153492	2.03051326	0.01237958	1.17476918
PE(18:0/20:4)-H	PE	C43 H77 O8 N1 P1	766.539231	11.5051781	1.55899537	0.02358573	2.91621258
PE(18:1/22:6)-H	PE	C45 H75 O8 N1 P1	788.523581	10.323258	1.98966059	0.02424517	1.17590614
PE(18:0/22:6)-H	PE	C45 H77 O8 N1 P1	790.539231	11.2313382	2.08883459	0.01561633	2.20426914
PI(16:0/18:2)-H	PI	C43 H78 O13 N0 P1	833.518557	9.29326139	1.56851322	0.01842781	1.53643448
PI(16:0/18:1)-H	PI	C43 H80 O13 N0 P1	835.534207	10.0911401	1.56876413	0.0223933	1.66545385

## Discussion

PCOS is a highly heterogeneous disease. In women with PCOS, the oocytes are often weak quality, which leads to lower fertilization, cleavage, implantation, and increased miscarriage rates [10]. The oocyte micro-environment provides the necessary requirements for oocyte developmental competence. FF is the liquid that surrounds the oocyte, forms its micro-environment and plays a key role in its development [3]. Therefore, it is crucial to explore the FF metabolite pattern. Untargeted metabolomics, which focuses on the dynamic changes of all small molecules in response to the disturbance of the organism, can provide deep insights for the etiopathogenesis and the discovery of biomarkers for various diseases [11]. Several researches of metabolomics on PCOS in plasma have found that the lipids were significantly changed. Lipidomics, a branch of metabolomics, is a high-throughput analytical technique that can systematically and efficiently analyze lipid composition and expression changes in various biological processes. At present, lipidomics analysis technology is generally based on liquid chromatography-mass spectrometry (LC-MS) platform and is mainly divided into untargeted and targeted analysis. Non-targeted lipidomics can systematically analysis of various types of lipids in the sample without bias, and the targeted lipidomics is mainly used for selective and specific quantitative analysis of specific lipids [12].

The present pilot study is the first to comprehensively investigate lipid profile changes in PCOS with normal weight in FF using the untargeted lipidomics approach based on ultra-high performance LC coupled with Q-Exactive MS. A number of lipids were detected in FF by this sensitive technique. We identified a series of differential lipids including triglycerides (TG), phosphatidylethanolamines (PE), phosphatidylinositols (PI) and etc. between women with PCOS and the healthy women.

TG was the only differentially represented subclass of lipids in FF of PCOS patients and health controls in this study (shown in Figure 4 of supplementary material). TG is a combination of three fatty acids combined with glycerol whichis the main source of energy. Patients with PCOS often have dyslipidemia mainly includes high levels of LDL and TG and low levels of HDL. Furthermore, lipid abnormalities are closely associated with obesity, insulin resistance and hyperandrogenemia in PCOS patients [13]. On the other hand, obesity has an important influence on the lipid metabolism [14]*.* Recently, a case-control study was conducted on 153 women with PCOS and 449 healthy women as controls to compare the serum lipid profile. Each group was divided into normal, overweight and obesity subgroups according to the BMI. Surprisingly, significant differences in TG were found only between obese women with PCOS and obese women without PCOS. There was no difference in plasma TG levels between the two groups of non-obesity women [15]. As refer to FF, Liu et al. found that a reduced level of TG was highly related to the lower fertilization rate in PCOS [16]. However, increased BMI is associated with elevated TG levels in ovarian FF [17]. This research on the lipid profile in FF was enrolled PCOS women with average weight heavier than normal women. To our knowledge, the lipid metabolite profiles in FF of PCOS patients and normal women with weight matched were not been investigated previously. In this study, significantly increased TG levels in FF were found in PCOS compared with normal women. Increased TG levels might be associated with low quality of oocyte in PCOS patients. A research found that mouse cumulus-oocyte complexes exposed to lipid-rich FF during their maturation had increased oocyte lipid content, induction of endoplasmic reticulum stress markers, and impaired oocyte nuclear maturation [17]. TG accumulation in the FF was also correlated to the levels of adipokines and proinflammatory cytokines in FF, implying inflammatory processes in the FF that are caused by high TG levels and may also attenuate oocyte development [18]. 

In this study, many species of PE showed higher accumulation in FF of the PCOS patients. Considering the potential functions of PE, it was found that PE is a major phospholipid class in the membranes of eukaryotic cells and modulates the membrane fluidity [19]. FF was centrifuged at 800 g which only pellet cells were removed but not organelles and vesicles. So the determined PE and PI may come from membrane structures of sub organelles or vesicles. PE also have been determined in FF by other lipidomic analysis. FF samples collected from patients who underwent IVF, including normal responder women who became pregnant (control group), women with PCOS and a hyper response to gonadotropins (PCOS group) and women with only hyper response to gonadotropins (HR group) were found that some form of PEs were higher represented HR groups and lower represented in PCOS group than the control group [20]. Regarding our study, although many forms of PE were high presented in PCOS patients, the total  PE level was found no significant difference between the two groups in this paper. 

Finally, phosphatidylinositol (PI) was found to be presented in high levels in the PCOS patients with normal weight. PI is composed of a glycerol backbone, two acyl chains esterified and an inositol ring linked by a phosphate. Although PI constitutes only 5–10% of total cellular lipids in mammalian cells, it is the source of seven phosphorylated derivatives of PI which play a major role in a vast array of cellular functions including signalling, membrane trafficking, ion channel regulation and actin dynamics [21]. The PI subclass of lipid has previously been detected in FF. Thais et al. compared MALDI-MS lipid fingerprints in the FF of young poor responder women in and normal responders. A lipid ion belonging to the PI subclass was overrepresented in the poor ovarian response group [22]. Another lipidomic analysis of FF samples revealed that some PIs were higher in normal responder women than in women with PCOS and a hyper response to gonadotropins and women with only hyper response to gonadotropins. In this study, some forms of PI were high presented in PCOS patients, but the total PI level was found no significant difference between the two groups. 

In this pilot study, lipid alterations of FF were found in PCOS patients with normal-weight, and those lipids might be considered as potential biomarkers of oocyte micro-environment in PCOS patients with normal-weight. However, there are some limitations. The results were obtained using a small set of samples because strict criteria were used to screen the patients enrolled in this pilot study. This experimental design was a biomarker discovery study in general and the sample was used as a screening set, and hence the prospective biomarkers proposed in this study need to be confirmed in an independent cohort as the validation set. This study revealed that the lipid profiles of normal-weight women with PCOS were different from those found in normal women. Furthermore, the potential lipid markers found in FF, highlighted by the relative increase in TG in the PCOS groups, contributed to improving the understanding of the molecular mechanisms involved in PCOS women without overweight. These biomarkers have demonstrated that the lipids are related to molecular processes in the normal-weight PCOS, such as inflammatory processes and endoplasmic reticulum stress in FF, which are caused by high TG levels and may impaired oocyte nuclear maturation. For this reason, PCOS patients always have the impaired oocyte development during ART. Therefore, FF lipid profile analysis is an important tool for identifying a panel of potential biomarkers because it reflects the ovarian microenvironment.

## Conclusions

In conclusion, this is the first study using the untargeted lipidomics technology based on HPLC-MS to analyze the lipid subclasses alterations in normal-weight PCOS focusing on the micro-environment of the oocyte. A pattern recognition technique allowed us to specifically discriminate normal-weight patients with from the normal women during IVF, which described a comprehensive picture of the lipid alterations that occurred in PCOS. The identified dysfunctional lipids of TG, PE and PI might serve as important diagnostic tools and are closely related to alteration in FF of PCOS with normal weight.

## Supplementary Information

**Additional file 1.**

## Data Availability

The datasets used and/or analysed during the current study are available from the corresponding author upon reasonable request.

## Abbreviations

POCS: Polycystic ovary syndrome
IVF: In vitro fertilization
COC: Cumulus-oocyte complexes
FF: Follicular fluid
BMI: Body mass index
ICSI: Intracytoplasmic sperm injection
FSH: Follicle-stimulating hormone
LH: Luteinizing hormone
E_2_: Estradiol
T: Testosterone
HDL-C: High-density lipoprotein cholesterol
LDL-C: Low-density lipoprotein cholesterol
AMH: Anti-Mullerian hormone
LDL: Low-density lipoprotein
HDL: High-density lipoprotein
TG: Triglycerides
PE: Phosphatidylethanolamines
PI: phosphatidylinositols
