# Supplementary material for: Lipidomics analysis of human follicular fluid form normal-weight patients with polycystic ovary syndrome: a pilot study
Source: J Ovarian Res. 2021 Oct 13;14:135. doi: 10.1186/s13048-021-00885-y (PMC8515674; doi:10.1186/s13048-021-00885-y)
Supplement: Supplementary file 1 — Additional file 1. [file 13048_2021_885_MOESM1_ESM.docx]

**Supplementary materials**

**A**

B

Fig.1. The base peak chromatogram of each QC sample in positive model (A) and negative model (B).


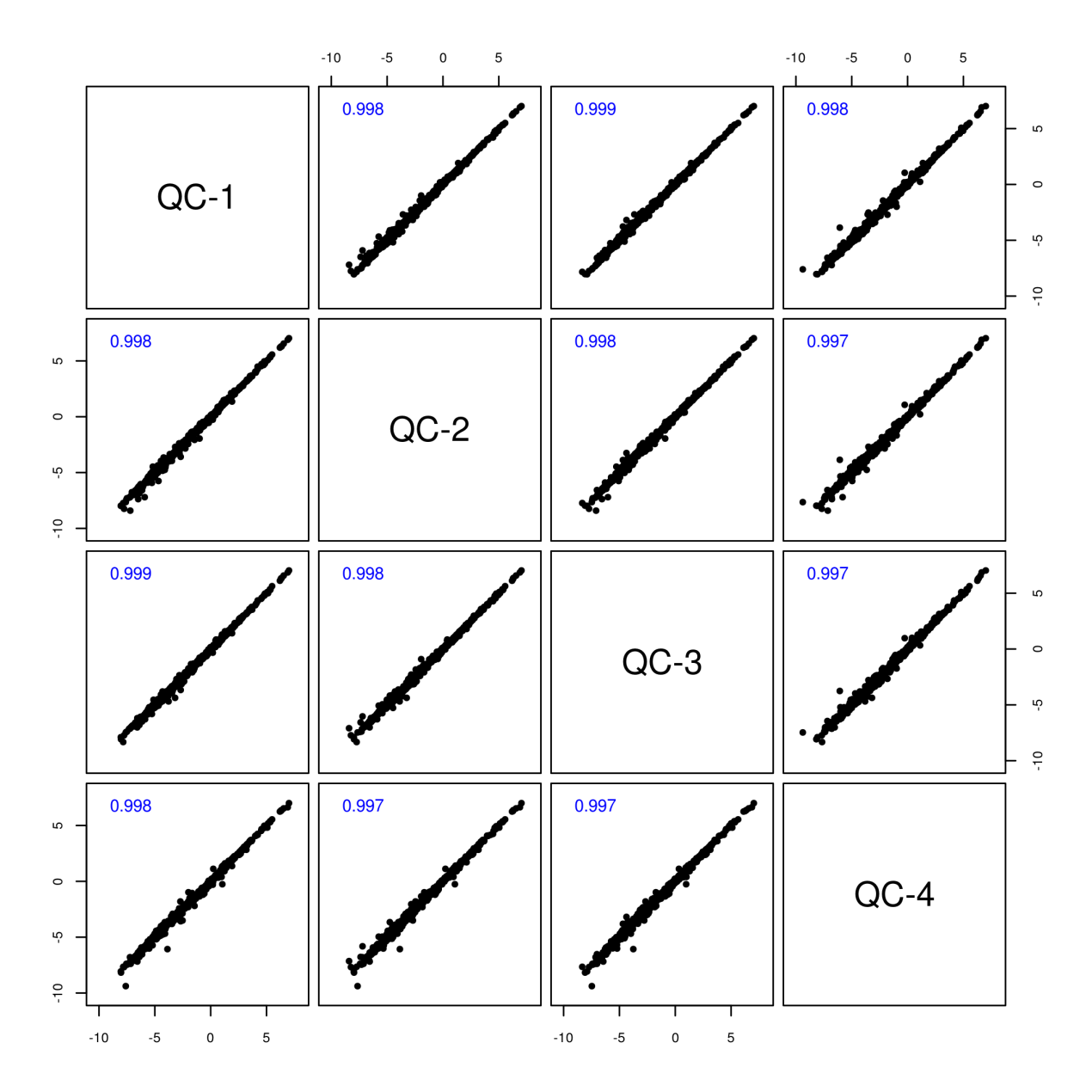


Fig. 2. The Pearson correlation analysis of the four QC samples based on the response intensity of the extracted peaks


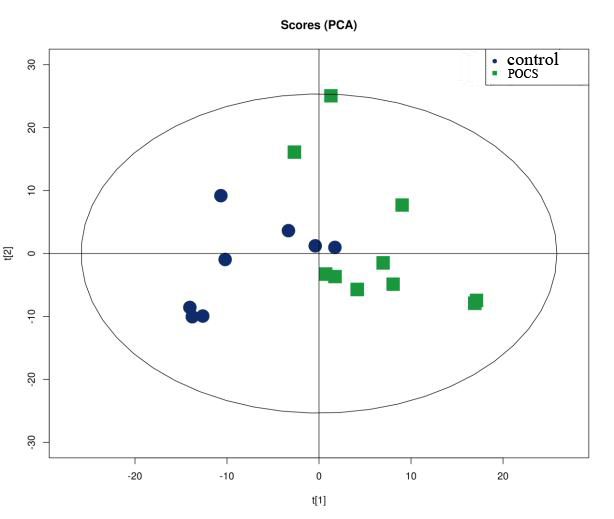


Fig. 3. Score plots of PCA model showed separating between normal weight PCOS women (blue circle) to control women (green square)

**P=0.007**

Fig. 4. Comparison of the lipid class of the PCOS group and the control group in follicular fluid

Table 1 The significantly differential lipids with VIP more than 1 and P < 0.05.

| **LipidIon** | **Class** | **IonFormula** | **CalMz** | **RT-(min)** | **Fold Change** | **P-value** | **VIP** |
| --- | --- | --- | --- | --- | --- | --- | --- |
| PE(16:0/22:6)+H | PE | C43 H75 O8 N1 P1 | 764.522484 | 10.2352508 | 2.1364297 | 0.01170255 | 1.2827738 |
| TG(16:0/14:0/18:1)+NH4 | TG | C51 H100 O6 N1 | 822.754516 | 20.7435549 | 2.82786013 | 0.00768395 | 1.04024184 |
| TG(16:1/16:1/18:1)+NH4 | TG | C53 H100 O6 N1 | 846.754516 | 19.714067 | 2.85935032 | 0.00103754 | 2.07802736 |
| TG(16:0/16:0/18:2)+NH4 | TG | C53 H102 O6 N1 | 848.770166 | 20.8812114 | 2.84645308 | 0.00753119 | 3.11342089 |
| TG(16:0/16:0/18:1)+NH4 | TG | C53 H104 O6 N1 | 850.785816 | 21.7834094 | 2.9164798 | 0.00508461 | 3.14637718 |
| TG(16:1/18:1/18:2)+NH4 | TG | C55 H102 O6 N1 | 872.770166 | 19.859873 | 2.51669633 | 0.00941933 | 4.83457578 |
| TG(16:0/18:1/18:2)+NH4 | TG | C55 H104 O6 N1 | 874.785816 | 20.8954062 | 2.26683859 | 0.00796182 | 7.04217237 |
| TG(16:0/18:1/18:2)+NH4 | TG | C55 H104 O6 N1 | 874.785816 | 19.8521076 | 2.42105627 | 0.00117044 | 1.21618584 |
| TG(16:0/18:1/18:1)+NH4 | TG | C55 H106 O6 N1 | 876.801466 | 21.7866945 | 2.51369634 | 0.00910841 | 6.11421082 |
| TG(18:0/16:0/18:1)+NH4 | TG | C55 H108 O6 N1 | 878.817116 | 22.584629 | 2.22068302 | 0.0275356 | 1.3333522 |
| TG(18:1/18:2/18:3)+NH4 | TG | C57 H102 O6 N1 | 896.770166 | 19.4825832 | 2.55170831 | 0.00642044 | 1.03093598 |
| TG(18:2/18:2/18:2)+NH4 | TG | C57 H102 O6 N1 | 896.770166 | 18.8771548 | 2.24487784 | 0.00535694 | 1.51541463 |
| TG(16:0/18:1/20:4)+NH4 | TG | C57 H104 O6 N1 | 898.785816 | 20.5420673 | 2.42182935 | 0.00972723 | 1.68243907 |
| TG(18:1/18:2/18:2)+NH4 | TG | C57 H104 O6 N1 | 898.785816 | 19.884727 | 2.00402129 | 0.00849049 | 2.6606902 |
| TG(18:1/18:1/18:2)+NH4 | TG | C57 H106 O6 N1 | 900.801466 | 20.9160682 | 1.83056246 | 0.01101498 | 3.33870883 |
| TG(18:0/18:1/18:2)+NH4 | TG | C57 H108 O6 N1 | 902.817116 | 20.8947311 | 1.52937154 | 0.03751919 | 1.62092888 |
| TG(18:0/18:1/18:2)+NH4 | TG | C57 H108 O6 N1 | 902.817116 | 21.8017114 | 2.30938518 | 0.01129426 | 3.83179623 |
| TG(16:0/18:1/20:1)+NH4 | TG | C57 H110 O6 N1 | 904.832766 | 22.5836836 | 2.22447076 | 0.02719304 | 1.57736708 |
| LPE(18:1)-H | LPE | C23 H45 O7 N1 P1 | 478.293916 | 2.96803702 | 1.35627658 | 0.04383678 | 1.31595936 |
| Cer(d18:1/16:0)+HCOO | Cer | C35 H68 O5 N1 | 582.510298 | 11.3804774 | 1.65187002 | 0.02982499 | 1.19827027 |
| PE(16:0/18:2)-H | PE | C39 H73 O8 N1 P1 | 714.507931 | 10.7122963 | 2.02509893 | 0.00623164 | 2.06847352 |
| PE(16:0/18:1)-H | PE | C39 H75 O8 N1 P1 | 716.523581 | 11.4653225 | 1.87622225 | 0.00103417 | 1.36389091 |
| PE(16:0/20:4)-H | PE | C41 H73 O8 N1 P1 | 738.507931 | 10.5345784 | 1.94530888 | 0.01049121 | 2.08653155 |
| PE(18:0/18:2)-H | PE | C41 H77 O8 N1 P1 | 742.539231 | 11.6753321 | 1.59339478 | 0.03091878 | 2.34017355 |
| PE(18:0/18:1)-H | PE | C41 H79 O8 N1 P1 | 744.554881 | 12.413823 | 1.66288434 | 0.00777766 | 1.2000378 |
| PE(16:0/22:6)-H | PE | C43 H73 O8 N1 P1 | 762.507931 | 10.2584914 | 2.08517498 | 0.01442886 | 3.08345178 |
| PE(18:1/20:4)-H | PE | C43 H75 O8 N1 P1 | 764.523581 | 10.6214751 | 1.71023624 | 0.0148648 | 1.68604552 |
| PE(16:0/22:5)-H | PE | C43 H75 O8 N1 P1 | 764.523581 | 10.9153492 | 2.03051326 | 0.01237958 | 1.17476918 |
| PE(18:0/20:4)-H | PE | C43 H77 O8 N1 P1 | 766.539231 | 11.5051781 | 1.55899537 | 0.02358573 | 2.91621258 |
| PE(18:1/22:6)-H | PE | C45 H75 O8 N1 P1 | 788.523581 | 10.323258 | 1.98966059 | 0.02424517 | 1.17590614 |
| PE(18:0/22:6)-H | PE | C45 H77 O8 N1 P1 | 790.539231 | 11.2313382 | 2.08883459 | 0.01561633 | 2.20426914 |
| PI(16:0/18:2)-H | PI | C43 H78 O13 N0 P1 | 833.518557 | 9.29326139 | 1.56851322 | 0.01842781 | 1.53643448 |
| PI(16:0/18:1)-H | PI | C43 H80 O13 N0 P1 | 835.534207 | 10.0911401 | 1.56876413 | 0.0223933 | 1.66545385 |
| PC(18:0/20:3)+HCOO | PC | C47 H87 O10 N1 P1 | 856.607311 | 11.6660913 | 1.20517005 | 0.03179569 | 1.9076139 |
| PI(16:0/20:4)-H | PI | C45 H78 O13 N0 P1 | 857.518557 | 9.13174946 | 1.32385449 | 0.04040975 | 1.36442594 |
| PI(18:0/20:3)-H | PI | C47 H84 O13 N0 P1 | 887.565507 | 10.5837271 | 1.4974899 | 0.03793427 | 1.34628739 |
